# The Application of Supercritical Fluid Extraction in Phenolic Compounds Isolation from Natural Plant Materials

**DOI:** 10.3390/molecules23102625

**Published:** 2018-10-12

**Authors:** Katarzyna Tyśkiewicz, Marcin Konkol, Edward Rój

**Affiliations:** Supercritical Extraction Department, New Chemical Syntheses Institute, Al. Tysiąclecia Państwa Polskiego 13A, 24-110 Puławy, Poland; marcin.konkol@ins.pulawy.pl (M.K.); edward.roj@ins.pulawy.pl (E.R.)

**Keywords:** anthocyanins, carbon dioxide, extraction techniques, flavonoids, phenolic compounds

## Abstract

The separation of phenolic compounds by supercritical fluid extraction has been widely studied throughout the last two decades. This is evidenced by a number of publications and articles. Supercritical fluid extraction (SFE) has become thus the effective method of separating the mentioned group of compounds. On the other hand, SFE is a beneficial approach in plant waste materials utilization and reduction of environmental burdens caused by the wastes. The aim of the study is to gather and systematize available information on the phenolic compounds separation that have been reported so far as well as to evaluate whether there is one optimal supercritical fluid extraction method for the phenolic compounds.

## 1. Introduction

The phenolic compounds are of a great interest for different institutions aimed at the applications of phenolics in food, cosmetics and pharmaceutical industries. Seeds, leaves, fruits and roots are the richest sources of not only polyphenols but also other groups of compounds, such as vitamins, fatty acids, triacylglycerols and others [1,2]. The efforts are made to obtain natural antioxidants and replace the synthetic ones that would be extremely beneficial from the viewpoint of health [3,4]. The natural products are characterized by thermal instability and high possibility of degradation during all kinds of processing steps [5]. As far as the phenolic compounds are concerned, a number of different extraction techniques has been introduced, with the most frequent being with the use of water and organic solvents (e.g., ethanol, methanol) [6,7]. Ethanol, water and supercritical carbon dioxide are all classified as generally recognized as safe solvents (GRAS) [8]. The choice of an extraction solvent is dependent on the properties of the extracted compounds. Koubaa et al. [9] provide an example of the extraction with hexane as a solvent, obtaining the extract characterized by low phenolic compounds content as a result of low solubility of the phenolic compounds in non-polar solvents. The phenolic compounds have been traditionally extracted using a Soxhlet method but also by soaking and stirring, heat reflux and maceration [10]. Vankar [11] proposed also steam distillation and hydrodistillation. The effectiveness of the extraction is assessed by the extraction selectivity, time, yield and especially the quality of the product obtained (extract) [12,13]. In the case of the phenolic compounds extraction, extraction method, chemical nature of compounds extracted as well as storage conditions are the crucial factors [14]. Several drawbacks of the conventional methods of the phenolic compounds extraction have been observed, such as low quality of an extract with unsatisfactory extraction yields but also economic unprofitability of the methods [7]. According to Panja [10], supercritical fluid extraction has been introduced as an efficient and cleaner method compared to other techniques.

**Figure 1:**
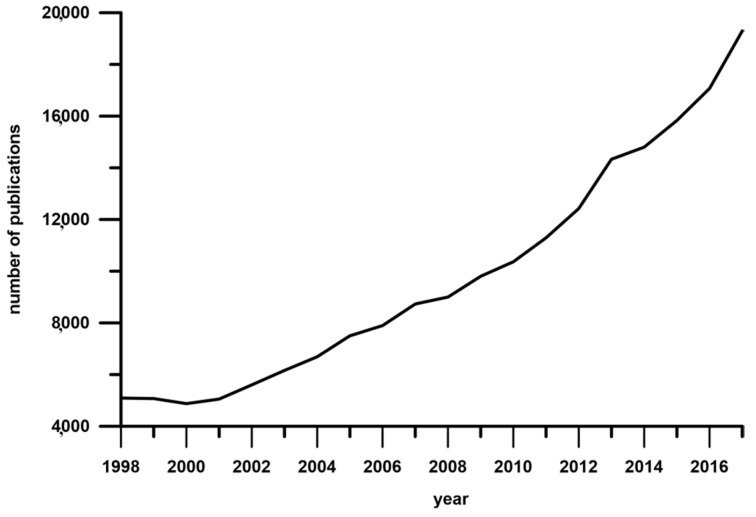
Evolution in the number of papers with the keyword “phenolic compounds” (Science direct, June 2018).

The interest in phenolic compounds has increased even 4-times in the last two decades. This is evidenced by a number of search results with the phrase “phenolic compounds” (Figure 1).

## 2. Phenolic Compounds

The phenolic compounds called secondary metabolites are a group of compounds widely spread in nature [15]. From a chemical point of view, polyphenols are comparable to alcohols in terms of a structure. Both groups of compounds possess hydroxyl groups –OH. However, in flavonoids they are attached to an aromatic ring [16]. The antioxidant properties of polyphenols are mainly attributed to their ability to prevent side reactions via free radicals neutralization [17].

Pereira et al. [16] distinguished two main groups of phenolic compounds, namely phenolic acids and flavonoids. The first group includes benzoic acids derivatives as well as cinnamic acid derivatives. The second group comprises low molecular weight compounds, called flavonoids, which are flavones, flavonols, flavanones, flavan-3-ols, anthocyanidins, isoflavones, coumarins, stilbenes, lignans. The sources of these compounds are leaves, seeds, bark and flowers [18,19]. The concentration of phenolic compounds may differ between the bark and leaves and fruits due to the differences in the exposition to light. As a result, polyphenols are synthesized to a higher extend in fruits which receive a higher dose of sunlight [20,21].

Phenolic compounds are interesting in terms of a broad spectrum of biological properties, including antidiabetic, antioxidant, gastroprotecitve, anti-inflammatory, spasmolytic, antimicrobial, anticarcinogenic [22,23] but also antiseptic, disinfectant [24], hepato-protective, hypotensive and cardioprotective [25]. They have an influence on chronical and degenerative diseases [2] and different types of cancer [26,27]. The presence of phenolic compounds in plant materials determines their use in various areas of cosmetic, food and pharmaceutical industries. Different plant materials represent different sources of particular groups of polyphenols. Peels and seeds are considered to be a good source of phenolic acids as well as flavonoids, especially polymethoxylated flavones and the glycosylated flavanones, found mainly in citrus fruits [28]. Berries contain such antioxidants as phenolic acids, flavonoids, hydrolysable and condensed tannins possessing health-promoting properties [29,30]. For instance, rutin is the most representative compound in, such materials as elderberry [31].

Some of the flavonoids as well as accompanying carotenoids affect the color of the obtained extract [32]. Rosemary is an interesting material in terms of characteristic phenolic diterpenes, among which are i.a. carnosol, carnosoic acid, rosmadial, rosmanol, epirosmanol, isorosmanol [33,34]. Extract of rosemary leaves is used in the food industry as a human nutritional ingredient. Moreover, the effect of extracts on cancer and neurodegenerative diseases has been investigated; [35,36,37]. The valuable group of anthocyanidins, including cyanidine-3-glucosides, malvidin-3-glucosides and peonidine-3-glucosides may be found in grape peels [38,39], whereas catechin, epicatechin, trans-resveratrol and procyanidin B1 are present in grape seeds [40]. The total content of polyphenols in grapes may be in the range of 60–70% (wt %) [41,42]. Apart from phenolic compounds, the plant materials contain also other compounds from the group of carbohydrates, proteins, vitamins and minerals, which are necessary from the nutritive viewpoint [43]. Pigeon pea leaves are known in traditional folk medicine, in which they serve as an agent with expectorant and analgesic properties. The most common compounds are stilbenes, cajaninstilbene acid and pinostrobin [44]. Some of the materials, for instance dandelion, are used as a medicine due to their antidiabetic, antirheumatic and diuretic properties [45,46]. Calycopterin belongs to a group of flavonoids present in a high amount in *Calyopteris floribunda*. It is responsible for anthelmintic and antiviral properties [47]. Among plant materials studied in terms of the phenolic compounds supercritical fluid extraction were *Maydis stigma* and *Pueraria lobata*. Both materials are used in Chinese medicine as medicinal herbs. It is due to the presence of alkaloids, saponins, tannins and flavonoids [5]. In case of the latter group, puerarin, daidzin, genistin, daidzein and genistein are identified in the high amount [48].

**Figure 2:**
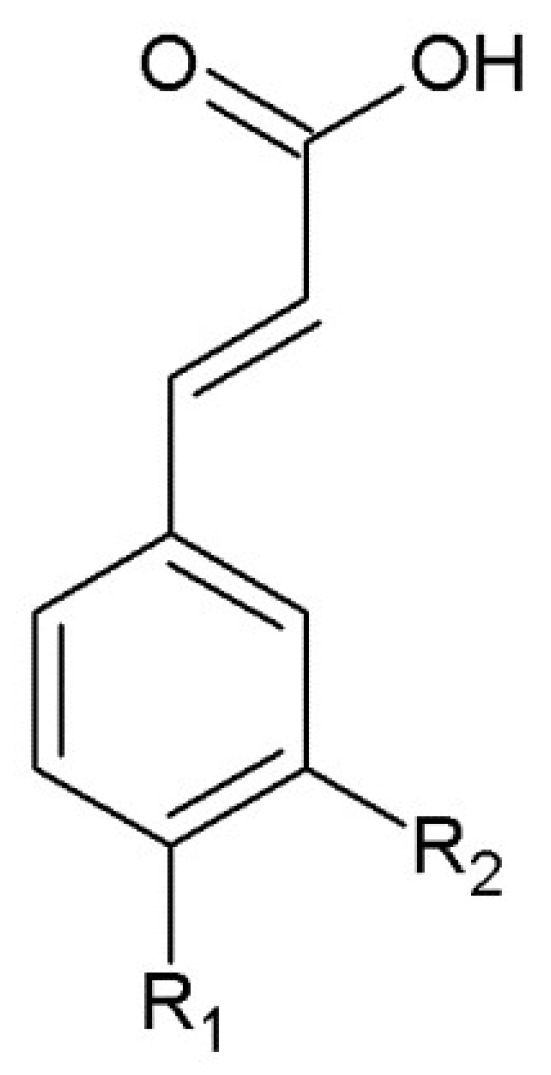
The structure of phenolic acids (R_1_=R_2_=H—cinnamic acid; R_1_=OH, R_2_=H—*p*-coumaric acid; R_1_=R_2_=OH—caffeic acid; R_1_=OH, R_2_=OCH_3_—ferulic acid).

The studies performed by Liakopolous et al. [49] indicated the presence in peach leaves, such compounds as phenolic acids (caffeic acid, chlorogenic acid, *p*-coumaric acid) (Figure 2), kaempferol, quercetin, isoquercetin and tannin, whereas luteolin, apigenin, luteolin-7-*O*-glucoside, apigenin-7-*O*-glucoside and chlorogenic acid are present in common yarrow (*Achillea millefolium*) [50]. In turn, poplar buds are characterized by the presence of compounds belonging to flavan-3-ols as well as other flavonoids, especially pinostrobin, pinocembrin, galangin and chrysin [51]. Such compounds as cyanidin, delphinidin, malvinidin, peonidin and petunidin are likely to be present in bilberry (*Vaccinium myrtillus*) [52]. Another interesting group of compounds are prenylflavonoids, with xanthohumol as the most common representative. It is worth mentioning that xanthohumol is present only in hops [53].

## 3. Low-Cost Sources of Phenolic Compounds

**Figure 3:**
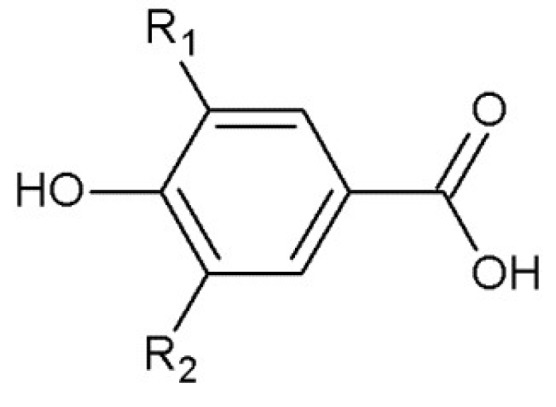
The structure of benzoic acid derivatives (R_1_=R_2_=H—hydroxybenzoic acid; R_1_=OH, R_2_=H—protocatechuic acid; R_1_=OCH_3_, R_2_=H—vanillic acid; R_1_=R_2_=OH—gallic acid; R_1_=R_2_=OCH_3_—syringic acid).

From the economical point of view, the valuable sources for the extraction of phenolic compounds are low-cost products as well as waste materials obtained by all kinds of preparations and industrial processing. One of materials currently of great interest are potato peels, which depending on a variety, contain functional compounds, including those with antioxidant properties [54]. Another example may be spruce bark waste as the by-product of wood processing and forest industry. It is a rich source of phenolic compounds in a form of benzoic acids derivatives (3,4- and 2,4-dihydroxybenzoicacids) (Figure 3) as well as flavonoids (quercetin, taxifolin) and cinnamic acids (ferulic acid, *p*-coumaric acid and sinapic acid) [55,56,57].

**Figure 4:**
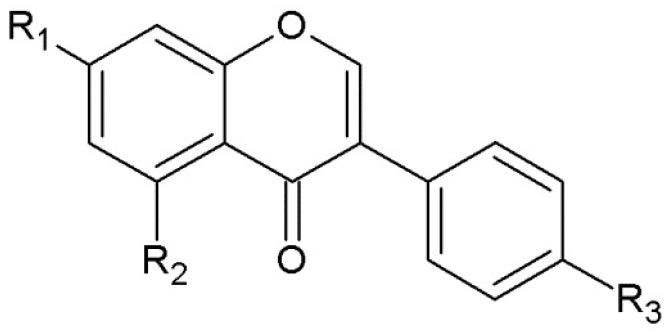
The structure of isoflavones (R_1_=R_3_=OH, R_2_=H—daidzein; R_1_=O-Glc, R_2_=H, R_3_=OH—daidzin; R_1_=R_2_=R_3_=OH—genistein; R_1_=O-Glc, R_2_=R_3_=OH—genistin).

Supercritical fluid extraction has found applications in solving problems incurred as a result of treatment and utilization of olive oil mill waste, which is considered as one of the most serious problems affecting environment [58]. Fruit juice production generates the solid waste streams, rich of valuable biologically active compounds, which are extracted with the use of supercritical fluid extraction [31]. The extraction of orange, pomelo, pomegranate by-products, including seeds and peels may give an opportunity to obtain fractions for such industries as food, cosmetic and pharmaceutical ones [27,59,60]. Seeds are also a valuable source of unsaturated fatty acids [28]. Wheat germ and soybean cake are by-products of flour milling and soybean oil processing, respectively [61,62]. In the case of soybean, it contains a group of phenolics, called isoflavones (Figure 4). They are attributed to have an influence on heart diseases and osteoporosis as well as postmenopausal symptoms [63,64,65,66,67].

## 4. Separation of Phenolic Compounds with Supercritical CO_2_ Extraction

Supercritical fluid extraction is often used for the extraction of non-polar compounds [12,66]. According to Liu et al. [5], it is an alternative technique to conventional extraction techniques, such as Soxhlet and other, owing its advantages to low density and viscosity of supercritical CO_2_ as well as high diffusivity. Moreover, carbon dioxide is non-flammable and “green”, thus it has no significant impact on the environment. The influence of the pressure on the solubility of desired compounds may increase the extraction efficiency. Moreover, carbon dioxide may be more selective when pressure and/or temperature is optimized [9,67]. The extraction with pure carbon dioxide provides the final product with no solvent as carbon dioxide is evolved and thus removed during depressurization [68].

### 4.1. Applications

#### 4.1.1. Pure Carbon Dioxide

The extraction of phenolic compounds from different plant materials with the use of carbon dioxide in a supercritical state has been studied in the temperature range 25–120 °C and pressure range 80–655 bar. 

Several authors studied the extraction of polyphenols in the temperature range 40–60 °C at different pressures of carbon dioxide [19,25,57,61,69,70,71,72,73,74,75,76,77]. Piantino et al. [69] used pure CO_2_ to extract antioxidant fractions from *Baccharis dracunculifolia* with optimal conditions of 60 °C and 400 bar, resulted in 2-times higher yield (4 mg/g) compared to the yield of methanolic and ethanolic extracts. The optimal extraction temperature for blackberry bagasse was also 60 °C [70]. The highest extraction yield (4.44 mg/g), which was obtained with 200 bar, was comparable to that reported by Piantino et al. [69]. The temperature of 60 °C and the pressure of 235 bar guaranteed the highest efficiency of supercritical fluid extraction for purslane (*Portulaca oleracea*) seeds [71]. In some cases the highest yield of the extraction does not correspond to the highest phenolic content. As it was studied by Chatterjee et al. [74], the temperature of 40 °C and the pressure of 350 bar resulted in the highest yield (8.51 mg/g) of *Phormidium valderianum* extraction. However, the content of phenolic compounds (55.98 mg GAE/g) was 2-times lower compared to the one in the extraction at the temperature of 50 °C and 350 bar (117.15 mg GAE/g). Comparing supercritical fluid extraction with the Soxhlet method, Bimakr et al. [19] achieved the same results for the extraction of spearmint leaves with two methods. The conditions for the SFE method were 60 °C and 200 bar, whereas the Soxhlet method was performed with 70% aqueous ethanol. The content of particular phenolics, including catechin, epicatechin, rutin, myricetin, luteolin, apigenin and naringenin was similar in both cases.

Depending on the extracted material, the antioxidant fraction was extracted at constant temperature of 40 °C and at different pressures, generally in the range 100–300 bar [41,50,76,78,79]. Only Feliciano et al. [66] performed the cranberries fruits extraction under the pressure of 655 bar. Carvalho et al. [76] and Wu et al. [79] performed studies on rosemary leaves and wine lees, respectively at the same conditions with the temperature of 40 °C and pressure of 300 bar. However, in the latter studies the extraction yield was 2-times higher (11.9% (*w*/*w*) vs. 5.0% (*w*/*w*)). The difference might be caused by the diversity of the phenolic compounds and their variable content in the extracted materials.

Concerning the number of publications, the extraction of rosemary (*Rosmarinus officinalis*) leaves to obtain phenolic compounds fraction was studied the most [33,37,75,76,80,81,82,83]. In this case, the studies were performed in the temperature range 25–110 °C and pressure range 80–1000 bar. Among all performed studies Nguyen et al. [84] used the highest pressures (500–1000 bar) for the extraction with pure CO_2_. In the first step, an essential oil fraction was extracted at the pressure of 115 bar and the temperature of 40 °C. In the second step, they reported optimal conditions for phenolic compounds extraction from rosemary leaves of 300 bar and the temperature in the range 90–100 °C as the further temperature increase was not efficient and enhanced thermal decomposition of the extracted compounds [84]. The temperature of 25 °C was not appropriate as it provided the total phenolic content of 120 mg per gram (as gallic acid equivalent) that was 2-times lower in comparison to the extraction performed at 50 °C under constant pressure of 80 bar. The optimal conditions in this case were the last mentioned (50 °C, 80 bar) [85]. Generally, the extraction may be reproducible when it is performed at similar conditions. Ivanović et al. [80] and Babovic et al. [33] reported rosemary leaves extraction at 100 °C and 300 and 350 bar, which resulted in the similar extraction yield of, respectively, 1.57% and 1.33% (*w*/*w*). Babovic et al. [33] extracted also sage (*Salvia officinalis*), thyme (*Thymus vulgaris*) and hyssop (*Hyssop officinalis*) at the same conditions with the extraction yields of 1.53%, 1.58% and 1.08% (*w*/*w*), respectively. Decreasing the temperature to 50 °C while increasing the carbon dioxide density at the constant pressure of 300 bar provides the best results in terms of the extraction yield (33%, *w*/*w*) of rosemary leaves extract compared to the results provided by the authors only as wt % [37].

Kuś et al. [51], Gelmez et al. [61] and Espinosa-Pardo et al. [73] performed similar studies in which they utilized supercritical fluid extraction with pure CO_2_ at the temperature of 60 °C (58 °C in [61]) and the pressure 300, 336 and 300 bar, respectively. In the case of peach (*Prunus persica*) fruits extraction, the conditions (60 °C, 300 bar) which enabled to obtain the highest yield of phenolic compounds (4.9 wt %) were different from the conditions for obtaining the highest yield of carotenoids (40 °C, 200 bar) [73]. In the case of *Strobilanthes crispus*, pomegranate (*Punica granatum*), mango (*Magnifera indica*), *Theobroma cacao*, *Undaria pinnnatifida* and bran, researchers performed the extractions with the highest yield in the temperature of 50 °C and in the pressure range 100–350 bar [77,81,82,83,86,87].

Another crucial parameter affecting the extraction of phenolic compounds is pressure. Studies performed by Wu et al. [45] indicated that increasing the pressure from 250 bar to 300 bar led to the increase of the cranberries herb extraction yield as well. However, further increase of the pressure above 300 bar decreased the efficiency of the extraction and consequently decreased the total yield. The pressure effect was explained by higher density of supercritical carbon dioxide at higher pressures. Kryževičiute et al. [88] investigated a similar influence of pressure on the phenolic extraction from raspberry pomace. At constant temperature of 60 °C, increasing the pressure from 100 bar to 450 bar resulted in 14-times higher extraction yield (from 0.01 wt % to 14.61 wt %). As it was pointed out by Wu et al. [45], the temperature increase lowers the density of carbon dioxide. However, Kryževičiute et al. [88] increased both the temperature and pressure to obtained optimal density of carbon dioxide to extract the phenolic compounds from raspberry (*Rubus* sp.) pomace with the highest yield. The lowest yield in the range 0.01–2.59% (*w*/*w*) was obtained when the pressure was 100 bar at each studied temperature (30, 45, 60 °C).

The phenolic compounds fraction was also obtained at higher temperature and pressure compared to previously described studies. The optimal conditions for grape seeds extraction were 104 °C and 538 bar [40]. Povilaitis et al. [89] performed the extraction of rye (*Secale cereale*) at the pressure of 550 bar with relatively low temperature (70 °C) than in the previous mentioned studies by Romabaut et al. [40].

The non-polar nature of carbon dioxide may be affected by the addition of a polar modifier. In the studies aimed at the comparison of the extraction of polyphenols with pure carbon dioxide and modified carbon dioxide, the most common modifiers were methanol, ethanol, water and isopropyl alcohol [54,57,90,91,92,93,94,95]. The influence of a modifier is best illustrated by the results of the research conducted by Leitao et al. [91]. The extraction of *Anacardium occidentale* with pure CO_2_ was compared with the extraction with CO_2_ with either 5% (*w*/*w*) isopropyl alcohol or 5% (*w*/*w*) ethanol at the temperature of 45 °C and pressure of 200 bar. The processes resulted in the extraction yield of 0.71% (pure CO_2_), 1.03% (CO_2_ + iPrOH) and 1.3% (CO_2_ + EtOH). However, the highest yield (1.6%) was obtained when the extraction was performed at lower temperature (35 °C) and lower pressure (100 bar) with 0.5% (*w*/*w*) ethanol. The authors compared also the supercritical fluid extraction method with conventional extraction method, such as Soxhlet with ethanol as a solvent. The yield (14.3 wt %) was over 10-times higher for the Soxhlet method in terms of the extraction yield when compared to a number of SFE methods with pure CO_2_, isopropyl alcohol and ethanol (0.7–1.6 wt %). De Azevedo et al. [92] performed the extraction of green coffee beans with pure CO_2_ as well as with 5% (*w*/*w*) of isopropyl alcohol or 5% (*w*/*w*) of ethanol as modifiers. The highest yield of 17% was obtained for the extraction with carbon dioxide and ethanol. The differences in using isopropyl alcohol and ethanol were observed in the selectivity and the detection of particular compounds. In the case of caffeine, its content was lower when the extraction was performed with isopropyl alcohol, whereas chlorogenic acid was not extracted with ethanol at all. Chang et al. [93] focused on the extraction of catechins from green tea at the temperature of 50 °C and pressure 310 bar.

Senorans et al. [75] provided the application of supercritical fluid extraction of rosemary leaves with the addition of 2% (*v*/*v*) ethanol at the temperature of 40 °C and pressure 300 bar. Cavero et al. [90] studied a higher range of the ethanol content (4%, 7% *v*/*v*) in the carbon dioxide mixture in the extraction of rosemary leaves. The differences between the extraction with pure carbon dioxide and modified carbon dioxide were significant in terms of the extraction yields, which were in the range 3.93–6.78% when ethanol was used. The pure carbon dioxide extraction yields were several times lower and amounted to 0.03–0.013%. Similarly, for the extraction of wine by-products, Louli et al. [95] employed supercritical fluid extraction with modified carbon dioxide. Methanol was used as a modifier in the amount of 5% (*v*/*v*). The extraction with methanol (45 °C, 250 bar) resulted in total phenolic content of 5.6% (*w*/*w*) and was 3 times lower than the extraction with pure CO_2_ at the temperature of 45 °C and the pressure of 150 bar. Two times higher extraction pressure (297.52 bar) and 44.39 °C were optimal in the sage herbal dust extraction as was reported by Pavlić et al. [96]. López-Padilla et al. [97] studied the influence of temperature (40, 70 °C) and pressure (200, 300 bar) on the *Vaccinium meridionale* extraction. Table 1 includes applications of pure carbon dioxide in phenolic compounds extraction from different plant materials. 

#### 4.1.2. Modified Carbon Dioxide

Carbon dioxide is characterized by its non-polar nature and it is said to be less capable of dissolving polyphenols, which are, on the other hand, polar compounds [13]. However, the extraction efficiency as well as solvent power of carbon dioxide may be increased by the addition of a polar co-solvent such as e.g., ethanol, methanol or water [66,100]. The addition of the co-solvent is based on the hydrogen bonds formation as well as dipole-dipole or dipole induced dipole interactions between co-solvent and an analyte but also affects the extraction by the solvent-co-solvent interaction [27,101]. Ivanović et al. [80] proved that the use of modifiers is efficient at particular pressures. In the case of the extraction of *Lamiaceae* herbs, the pressure of 500 bar and higher affected the selectivity of carbon dioxide resulting in a decrease of the antioxidant activity. Table 2 presents the applications of co-solvent modified carbon dioxide in phenolic compounds extraction.

The studies performed with the use of the supercritical fluid extraction may be grouped into five methods based on a type of a modifier: (a) pure and aqueous ethanol, (b) water, (c) sequential extraction with carbon dioxide, ethanol and water, (d) methanol, (e) other solvents (hexane, ethyl acetate, ethyl lactate, isopropyl alcohol). The most frequent extractions were performed with carbon dioxide and ethanol, either aqueous or pure [2,4,13,27,39,47,59,68,86,106,139]. The studies included the temperature range 20–80 °C, pressure range 80–600 bar as well as a different content of ethanol in carbon dioxide/ethanol mixtures. Manna et al. [2] extracted polyphenols from grape pomace at the temperature of 40 °C and the pressure of 200 bar. The content of ethanol was investigated in the range 5–25% (*w*/*w*). The results showed no improvement in the extraction yield with the increase of the ethanol content. Similar studies were performed by Ghaffor et al. [39] on grape peels with the optimal content of ethanol being ca. 6–7%. Different results were obtained in the studies by Adil et al. [4] and Kazan et al. [139]. In the first studies, the addition of 20% (wt %) ethanol was required to obtain the highest total phenolic content (0.427 mg GAE/g for apple pomace and 0.25 mg GAE/g for peach pomace). The optimal extraction conditions were 50 °C and 600 bar. Kazan et al. [139] applied lower pressure of 150 bar and 60 °C in the extraction of peach (*Prunus persica*) leaves. The extraction with 6% (wt %) ethanol yielded the phenolic content of 79.92 mgGAE/g. In a comparison, 30.66 mgGAE/g were obtained when 20% (wt %) ethanol was used with carbon dioxide.

Some of the authors performed the extraction at the temperature of 40 °C and in the pressure range 150–500 bar with pure ethanol content ranging from 0 to 15% (*w*/*w*) [13,68,110,118,121,125]. In both studies by Farias-Campomanes et al. [13,68], the same conditions were employed that resulted in the highest extraction yield of grape bagasse and lees (40 °C, 200 bar, 10 wt % ethanol). In both cases, increasing the pressure to 400 and 350 bar, respectively, decreased the efficiency even 2-times. The same amount of ethanol (10 wt %) guaranteed the highest yield of *Citrus paradisi* extraction (58.6 °C, 95 bar) in the studies by Giannuzzo et al. [110]. The mixture of carbon dioxide with 7.5% of ethanol was also used in the studies by Pascual-Marti et al. [121] and less than 7% of ethanol in studies by Ghafoor et al. [118] on the extraction of grape skin and seeds, respectively. Fiori et al. [125] used the highest pressure of 500 bar with the temperature of 45 °C to extract grape by-products.

The best results for the extraction (50 °C, 350 bar) of green propolis were reported by Machado et al. [127], using CO_2_ with a relatively low content of ethanol (1%). A different effect of processing parameters on *Undaria pinnatifida* seaweed extraction of phenolic compounds was also observed by Roh et al. [86]. The best conditions for obtaining the fucoxanthin rich fraction were 50 °C and 200 bar, whereas phenolic compounds rich fraction was obtained at 60 °C and under 250 bar. The authors used the mixture of carbon dioxide and 3% (*v*/*v*) ethanol. As for the extraction of green algae, the optimal conditions were 40 °C, 300 bar and carbon dioxide with 11.4 wt % ethanol [126]. However, the fraction with the highest polyphenols content was obtained at different conditions, involving 60 °C, 300 bar and 7.5% of ethanol as was demonstrated by Fabrowska et al. [126]. Valandez-Carmona et al. [148] reported studies on *Theobroma cacao* extraction in which conditions were provided (60 °C, 300 bar) that resulted in both highest extraction yield (0.47%) and at the same time highest total phenolic content as compared, for instance, with studies on green algae by Fabrowska et al. (60 °C, 300 bar) [126].

In some cases, the temperature did not influence the extraction of the phenolic compounds. Ouédraogo et al. [135] obtained the highest total phenolic content (247.78 mg GAE/g) at 55 °C and 250 bar in *Odontonema strictum* leaves extract. Higher temperature (65 °C) resulted in a lower content of the polyphenols (121.99 mg GAE/g). Liu et al. [27] also investigated the effect of the temperature on the polyphenols extraction from *Acanthopanax senticocus*. The optimal conditions involved the temperature range from 35 to 50 °C. The increase of the pressure between 320–340 bar also increased the extraction yield to some extent. However, as stated by the authors, such conditions influence more the financial aspect of the extraction. Pinelo et al. [123] compared the extraction of grape pomace under the same conditions (50 °C, 350 bar) with pure CO_2_ and modified CO_2_ (with 8% of ethanol). In this case, ethanol did not lead to the highest content of phenolic compounds. The extraction with pure CO_2_ resulted in the polyphenols concentration of 572.8 ppm, whereas 432.5 ppm with modified CO_2_. Similar studies were reported by Benelli et al. [59]. The ethanol content was the same, while the orange (*Citrus sinensis*) extraction was conducted at the pressure of 250 bar, which in this case resulted in more than 2 times higher extraction yield (3.0% *w*/*w*) than that obtained with pure carbon dioxide. Casas et al. [124] performed studies aimed at obtaining the resveratrol-rich fraction from grape pomace at the temperature of 55 °C and under the pressure of 100 bar without and with a modifier (5%, *v*/*v* ethanol). At the same conditions, resveratrol was not identified in the fraction obtained with pure carbon dioxide. Da Porto et al. [119] studied different concentrations (7.5, 10, 15%) of ethanol/water mixtures (57:43, *v*/*v*). The highest grape marc extraction yields were obtained when 7.5% of the mixture was used at 40 °C and 80 bar. It was observed that with a higher concentration of ethanol/water mixture (10%), the extraction yield was slightly lower (2527 mg GAE/100 g) than that with 7.5% of the mixture (2600 mg GAE/100 g). The highest content of ethanol (25–30%) in carbon dioxide/ethanol mixture was used by Bleve et al. [122]. The grape skins extraction was performed under liquid as well as subcritical conditions, which enabled to determine the optimal temperature between 30–40 °C. Zachova et al. [149] reported the addition of 20% (*v*/*v*) ethanol as optimal in the extraction of grape cane at the temperature of 50 °C and the pressure of 300 bar.

Another method has been applied for the extraction of antioxidants from *Solanum stenotomun*. The temperature of 65 °C and pressure of 400 bar with 5% (*v*/*v*) ethanol enabled to obtain the yield of 1%, which was the highest yield of the extraction. The highest content of anthocyanins was obtained at the pressure of 100 bar [54]. The highest yield (30.46%, *w*/*w*) for the extraction of spruce bark wastes was obtained using supercritical fluid extraction with carbon dioxide with the addition of the ethanol. The temperature was 40 °C and pressure 100 bar. However, the highest total phenolic content was reached when the extraction was performed at higher temperature of 60 °C. While the extraction was studied in the temperature range 40–60 °C, in each case the phenolic compounds were extracted within first 15 min [57]. The highest extraction yield (7.54%) was obtained from *Calyopteris floribunda* leaves when 5% (mass%) of ethanol was added to carbon dioxide at the temperature of 35 °C and 300 bar [47]. Other plant materials, such as gingko (*Gingko biloba*) [114], *Bupleurum kaoi* [107], *Momordica charantia* [134], blackberry (*Syzygium cumini*) [106] as well as eucalyptus [113] were studied in the temperature range 35–60 °C and the pressure range 50–350. The highest content of flavonoids (15 mg/g) was analyzed by Shan et al. [134]. The combination of carbon dioxide and 5% of aqueous solution of ethanol (EtOH/H_2_O: 25:75, *v*/*v*) provided the highest amount of polyphenols (7.31 mg/g as catechin equivalent) in the extraction of bamboo (*Sasa palmata*) leaves at the temperature of 95 °C and the pressure of 200 bar [104].

Some of the researchers studied the influence of carbon dioxide with 50% aqueous solution of ethanol [105,111,131] and even 60% aqueous solution of ethanol [147]. Del Pilar Garcia-Mendoza et al. [131] as well as Kerbstadt et al. [105] applied the acidified mixture of ethanol and water in the extraction of jucara (*Euterpe edulis*) and bilberry (*Vaccinium myrtillus*) press cake extraction, respectively. In the studies performed by Vatai et al. [111], the optimal conditions which guaranteed the highest total phenolic content of 60.6 mg/g were 40 °C and 150 bar. The authors investigated the effect of the temperature increase from 20 to 60 °C, which resulted in a different amount of anthocyanins. In the studies reported by Li et al. [147] on the extraction of tea (*Camelia sinensis*) seed cake, the extraction was conducted at the temperature of 80 °C with 60% aqueous ethanol. The pressure of 200 bar was required to obtain the satisfactory extraction efficiency. In a comparison with the studies performed by Li et al. [147], Woźniak et al. [109] performed the extraction with carbon dioxide and ethanol (20, 50, 80%, *m*/*m*) as a modifier. The optimal conditions for the Chokeberry (Aronia melanocarpa) extraction were 35 °C, 100 bar and 80% (*m*/*m*) of ethanol. The temperature of 35 °C was also optimal in the soybean expellers extraction conducted by Alvarez et al. [145]. However, higher pressure (400 bar) was required for obtaining the highest extraction yield.

The studies were also performed with the water content of 30% and less in ethanol/water mixtures [44,60,128,129,130,131]. The extractions were performed in the temperature range 35–80 °C and pressure range 220–390 bar. C.M. Liu et al. [132] combined carbon dioxide with 70% (*v*/*v*) of aqueous ethanol with the same pressure of 300 bar but different temperature, including 35 °C. Moreover, Li et al. [147] compared 70% aqueous ethanol and pure ethanol as a modifier. In this case *Koreanum nakai* leaves extraction yield for 70% (*v*/*v*) ethanol was 5.4%, whereas 3.4% was obtained with pure ethanol. The mixture of ethanol/water with the content of ethanol of 85% (*v*/*v*) was effective in the extraction of polyphenols from pomelo (*Citrus grandis*) peels at different temperatures, with the highest one (80 °C) being used by He et al. [60]. Roseiro et al. [150], He et al. [130], Escobedo-Flores et al. [136] and Kong et al. [44] employed the extractions at following conditions 40 °C/220 bar (carob (*Ceratonia siliqua*)), 50 °C/250 bar (hops (*Humulus lupulus*)), 55 °C/380 bar (oats (*Avena sativa*)) and 60 °C/300 bar (pigeon pea (*Cajanus cajan*)), respectively. All studies were performed with 80% (*v*/*v*) aqueous ethanol. Paes et al. [20] used 90% carbon dioxide and both 5% of ethanol and 5% of water to extract blueberry (*Vaccinium myrtillus*) wastes. The authors observed that different compositions of carbon dioxide/co-solvent mixture were required for highest extraction yield (50% CO_2_ + 50% H_2_O) and highest total phenolic content (90% CO_2_ + 5% EtOH + 5% H_2_O). Both studies were performed at the temperature of 40 °C and the pressure of 200 bar. Da Porto et al. [120] optimized the extraction conditions for the polyphenols extraction from grape marc with scCO_2_ + 15% H_2_O (*w*/*w*) and scCO_2_ + 15% EtOH (*w*/*w*). As for the first method, the temperature and pressure were 40 °C and 100 bar, whereas for the second 50 °C and 100 bar. However, the best results were obtained when the combined extraction was applied. The first step was to extract grape marc with CO_2_/H_2_O with the same concentration as earlier followed by CO_2_/EtOH. The extraction resulted in a total phenolic content of 733.6 mg GAE per 100 g of the material. In the consecutive studies, da Porto et al. [108] employed carbon dioxide with much lower content of ethanol (10%) in the ethanol/water mixture in the extraction of grape marc. In this case, such conditions with the temperature of 40 °C and the pressure of 80 bar enabled to obtain best results.

In some cases, the use of supercritical fluid extraction may not be limited only to extract phenolic compounds. In the studies by Stevenson et al. [151], they did not separate phenolic compounds from oats (*Avena sativa*) by supercritical fluid extraction. They applied SFE as the pre-treatment stage, in which the carbon dioxide may be used to remove the lipids from the sample at the temperature of 85 °C and pressure of 689 bar and prepare the sample in this way for further separation. The oats (*Avena sativa*) bran was then extracted with 50% aqueous solution of ethanol and pure ethanol. The highest extraction yield (9.2 mg/g) was slightly higher than that obtained with pure ethanol (9.0 mg/g).

The extraction with carbon dioxide modified by water may be the next option for the extraction of phenolic compounds [23,128,146]. Huang et al. [128] extracted powdered green tea and obtained the extracts which were characterized by the highest content of catechins and at the same time the highest content of caffeine. The optimal temperature and pressure were 60 °C and 300 bar, respectively. Similar results were reported by Veggi et al. [23]. The extraction of jatoba (*Hymenaea courbail*) bark was performed in the temperature range 50–60 °C and pressure range 150–350 bar. Leal et al. [146] optimized conditions for the extraction of sweet basil leaves for different addition of water (1, 10, 20 wt %) to carbon dioxide. As for 1% (wt %) of water, the temperature was 30 °C and the pressure was 100 bar. For 10% (*wt* %) of water, 30 °C and 100–150 bar were the optimal conditions, whereas 20% (wt %) of water required the temperature of 30 °C but a higher pressure of 300 bar compared to previous conditions. Babova et al. [52] tested three solvent mixtures at the temperature of 45 °C and the pressure of 250 bar. Carbon dioxide was modified by 6% (*w*/*w*) of the ethanol/water mixtures with ethanol content of 70% and 50% as well as 9% (*w*/*w*) of the ethanol/water mixture with ethanol content of 10% (*w*/*w*). The latter were the best conditions for the extraction of polyphenols from bilberry (*Vaccinium myrtillus*).

Da Porto et al. [108] indicated that the best results were obtained when the extraction was performed in sequential steps. Such studies were also performed by other groups of researchers [18,52,102,103,133,141,142,143]. The studies were aimed at the extraction of particular materials first with pure carbon dioxide, then the residue extraction with the mixture of ethanol and water and finally the residue extraction from step 2 with water. In both extraction methods proposed by Paula et al. [102,103], the extracted material was *Arrabidaea chica* leaves. In the first study, the process was applied with two-step extraction (CO_2_, CO_2_ + EtOH + H_2_O), whereas in the second one pure CO_2_ was followed by CO_2_ + EtOH and CO_2_ + H_2_O as two separate steps. Monroy et al. [141,142] applied in their studies on purple corn (*Zea mays*) three-step extraction (CO_2_, CO_2_ + EtOH, CO_2_ + H_2_O). Among studied temperatures (36–64 °C) and pressures (259–541 bar), the optimal conditions were 65 °C and 440 bar based on the response surface methodology. Del Pilar Sánchez-Camargo et al. [143] used two-step extraction of rosemary leaves with pure carbon dioxide and then with addition of 7% of ethanol, which enhanced the extraction efficiency. The extractions were performed at the temperature of 40 °C and 100 bar (only pure carbon dioxide) as well as 300 bar (pure carbon dioxide, carbon dioxide with ethanol). Bitencourt et al. [133] added one more step in the extraction of *Melia azedarach* fruits. The process was performed with pure carbon dioxide and then with carbon dioxide and ethanol followed by pure ethanol and finally with water.

Methanol is the third solvent that has been used commonly as carbon dioxide modifier. Different concentrations of methanol (0–40%, *v*/*v*) as well as aqueous methanol were studied (70, 80%) [17,34,66,95,115,116,117,137,138,140,144]. In the case of temperature and pressure, studies included the ranges of 35–100°C and 100–646 bar, respectively. The highest temperatures of 80 °C and 100 °C were applied by Khorassani et al. [117], Le Floch et al. [137] and Celiktas et al. [34] to extract phenolic compounds fraction from grape seeds, olive leaves and rosemary leaves, respectively. The optimal methanol concentration was 40%, 10% and 5%, respectively. Grape seeds were extracted with the highest pressure of 646 bar among all studies performed with carbon dioxide and methanol [117]. The highest methanol concentration of 40% was also effective in the extraction of grape wastes with relatively low temperature (35 °C) and pressure (103 bar) [17]. Phenolic compounds were not detected in the fractions obtained with pure carbon dioxide (35 °C, 100 bar as well as 45 °C, 200 bar). The addition of 5% methanol to carbon dioxide at the temperature of 45 °C and the pressure of 100 bar resulted in the same effect as with pure carbon dioxide. The increase of methanol to 15% and the pressure to 350 bar increased also the pistachio (*Pistachia vera*) extraction efficiency to the highest total phenolic content (7.8 mg tannic acid/g) level [140].

The concentration of methanol has also an effect on particular compounds extraction from grape seeds. The lowest concentration (2%, *v*/*v*) enabled to extract low molecular compounds. However, the increase of the concentration to 10% (*v*/*v*) resulted in the identification of epicatechin gallate in the obtained fractions [116]. Palma and Taylor [115] and Louli et al. [95] indicated 5–10% (*v*/*v*) of methanol as the one which provides the best results in the extraction of grape seeds and grape by-products. Louli et al. [95] performed extraction of grape by-products at the temperature of 45 °C and under 250 bar. The same conditions (45 °C, 250 bar) were used by Peng et al. to extract *Patrinia villosa* [138]. Both Lin et al. [144] and Zuo et al. [66] combined carbon dioxide with 70% and 80% aqueous methanol, which provided the best extraction yield in the extraction of *Scutellariae radic* and soybean, respectively.

**Figure 5:**
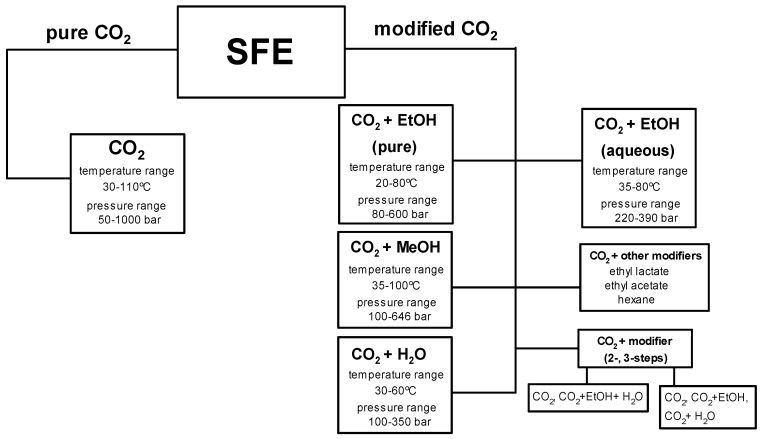
Application of SFE in phenolic compounds extraction from plant materials.

Apart from ethanol, water and methanol, which are the most commonly used as modifiers, other solvents were also tested, such as ethyl acetate, ethyl lactate and hexane [26,46,112,129]. Ethanol occurred to be better solvent than ethyl acetate and water in the studies on eucalyptus (*Eucalyptus globulus*) by Santos et al. [112] and guava (*Psidium guajava*) by Castro-Vargas et al. [46]. Different results were reported by Bermejo et al. [129]. In their studies, extraction of green tea leaves with carbon dioxide and ethyl lactate resulted in the highest caffeine yield. Abbasi et al. [26] indicated water as carbon dioxide modifier which provided the largest amount of phenolic compounds in the fraction obtained from pomegranate (*Punica granatum*). Hexane was the one which provided the poorest extraction efficiency. Figure 5 presents a schematic diagram of phenolic compounds extraction from various plant sources using pure and modified carbon dioxide.

## 5. Combined SFE

Supercritical fluid extraction (SFE) has found an application in the phenolic compounds extraction with the use of pure carbon dioxide as well as carbon dioxide with an addition of either polar or non-polar modifiers. Additionally, several other techniques (both extraction and chromatographic) have the potential to be used for obtaining fractions rich in polyphenols. Some of the used combinations are SFE-SFC (supercritical fluid extraction–supercritical fluid chromatography) [152,153], SFE-HPLC-MS/MS (supercritical fluid extraction–mass spectrometry liquid chromatography) [154], SFE-GC (supercritical fluid extraction-gas chromatography) [155], SFE-FTIR (supercritical fluid extraction–fourier transform infrared spectroscopy) [156], SFE-SPE (supercritical fluid extraction–solid phase extraction) [152], SFE-PLE (supercritical fluid extraction–pressurized liquid extraction) [106] but also US-SFE (ultra sound assisted supercritical fluid extraction) and SFE-DLLME (supercritical fluid extraction–dispersive liquid-liquid microextraction) [157].

Concerning the SFE-SFC system, both solvent in SFE and mobile phase in SFC are combined in the same physical state in order to extract, fractionate as well as identify and quantify desired compounds [152]. A successful application of SFE-SFC-MS/MS has been reported by Liu et al. [153] with the optimal extraction conditions of 50 °C and 9 minutes in an off-line mode. The extraction of 15 phenolic compounds from garlic required the addition of 30% methanol. The chromatographic separation was conducted with the use of Shim-pack UCX Diol (150 mm × 4.6 mm; 3 µm) column packed with silica modified by diol groups as well as carbon dioxide and methanol (with ammonium formate) as the mobile phase components. The obtained SFE fractions were rich in ferulic acid, *p*-coumaric acid, naringenin, apigenin, protocatechuic acid, isorhamnetin, luteolin, phthalic acid, quercetin, chlorogenic acid and resveratrol. As it was indicated by Hofstetter et al. [158], the use of on-line SFE-SFC is limited by the analysis duration as well as sample transfer performed in a split mode in order to reduce the extraction volume, which in consequence has an influence on final results and sample amount. Another interesting chromatographic technique coupled to supercritical fluid extraction was used by Hawthorne et al. [155]. The SFE-GC-FID was applied in the extraction and identification of phenolics from hardwood smoke with the extraction temperature of 50 °C and the pressure of 400 bar.

As far as SFE coupled to other extraction techniques is concerned, Zachová et al. [106] compared SFE with PLE (pressurized liquid extraction) method. Moreover, combined SFE-PLE system was applied to stilbenes extraction from grape cane. The conditions of PLE method for resveratrol isolation involved the temperature of 100 °C and the pressure of 100 bar, whereas SFE method required 60 °C and 300 bar with the addition of ethanol (15–20%) as a modifier. The obtained SFE-PLE fractions were characterized by different concentrations of stilbenes depending on the extraction parameters. As it was concluded by the authors, further optimization is required in terms of pressure and temperature in SFE method and the composition of the solvent or solvents mixtures in PLE method [106].

## 6. SFE among Other Green Extraction Methods

The advantages of SFE lie in the health and safety but also cost and environmental impact in general. The properties of carbon dioxide are particularly influential when thermolabile compounds are to be extracted [159]. Moreover, as it was discussed in this paper, SFE is an effective system for waste materials management not only reducing wastes but also reducing solvent and energy consumption. In addition to SFE, other modern green extraction techniques include ultrasound-assisted extraction (UAE), microwave-assisted extraction (MAE), pressurized liquid extraction (PLE) and pressurized hot water extraction (PHWE) [15,160]. Panja [10] adds also other techniques, such as enzyme assisted extraction (EAE) and pulsed electric field extraction (PEF), whereas sub-critical water extraction (SCWE) and high hydrostatic pressure processing (HHPP) are the techniques proposed by Khoddami et al. [161].

Rodríguez-Solana et al. [162] compared SFE and MAE in terms of the total phenolic content in the extracts of *Mentha piperita*, *Origanum vulgare*, *Rosmarinus officinalis* and *Thymus vulgaris*. The SFC method was used with carbon dioxide as well as methanol (3%, *v*/*v*) as a modifier, however, higher TPC value was obtained by ASE method in all four extracts (4.09, 3.04, 1.67 and 0.42 g GAE/100 g dry plant for ASE vs 0.02, 0.13, 0.11 and 0.07 g GAE/100 g dry plant for SFE). The comparison of SFE and MAE was also performed by Llopmart et al. [163] on the example of the phenolic compounds extraction from various soil matrices. The obtained results indicated a higher recovery for garden soil (111%) and endesa soil (85%) extraction in the case of MAE method. The garden soil and endesa soil extraction with the use of SFE resulted in the recovery of 88% and 77%, respectively.

Roseiro et al. [108] have reported the superiority of SFE over UAE method in the extraction of carob (*Ceratonia siliqua*) pods. The UAE method was performed with two solvents, such as 100% H_2_O and 70% acetone. The higher TPC was analyzed when acetone was used as a solvent (20.4 mg GAE/g vs 9.4 mg GAE/g). The SFC was conducted at the temperature of 40 °C and pressure of 220 bar with the addition of 10% EtOH/H_2_O (80:20, *v*/*v*). TPC in the obtained extract was measured in the amount of 27.1 mg GAE/g. In the studies performed by Dias et al. [164], SFE combined with US appeared to be more effective in the phenolic compounds extraction of pepper (Capsicum baccatum) in a comparison with a single SFE. The optimal conditions for SFE-US method were 40 °C, 150 bar and 200 W. The TPC value obtained for SFE (60 °C, 250 bar) extract was 3 times lower than for SFE-US extract (0.23 mg EAG/g *vs* 0.62 mg EAG/g). Benelli et al. [59] obtained similar TPC for SFE and UAE in the extracts from orange (*Citrus sinensis*). The values of TPC were 30 mg GAE/g and 37 mg GAE/g, respectively for SFE and UAE. The temperature of 50 °C and pressure of 250 bar with carbon dioxide modified by ethanol (8%, *v*/*v*) were the optimal conditions for the highest extraction yield. Among studied MAE solvents, such as hexane, ethyl acetate, ethanol, water and dichloromethane, the latter was the most effective.

A number of publications on the comparison of SFE with PLE method, has been reported by Taamalli et al. [164], Anaëlle et al. [165], Kraujaliene et al. [166] and Fernández-Ponce et al. [167]. In first two studies, better results were obtained for the PLE method. The global extraction yields of mango leaves were 37% and 8%, respectively for PLE and SFE [167]. In the case of PLE method proposed by Anaëlle et al. [165], different compositions of ethanol/water mixtures (EtOH/H_2_O, 75:25, *v*/*v*; EtOH/H_2_O, 25:75, *v*/*v*) did not significantly influence TPC values, which were around 10% of dry weight. SFE at the temperature of 60 °C and pressure of 152 bar with the addition of 12% (*v*/*v*) ethanol resulted in 2 times lower TPC (around 5% of dry weight). In some cases, SFE and SFE-PLE provide similar TPC values as was proved by Kraujaliene et al. [166] on the example of goldenrod (*Solidago virgaurea*) extraction with TPC around 169–185 mg GAE/g. The combination of acetone with either 70 °C or 140 °C in PLE and carbon dioxide with 70 °C in SFE-PLE method were used. Taamalli et al. [168] extracted six different Tunisian olive leaves with the use of SFE (40 °C), MAE (methanol/water, 80:20, *v*/*v*; 80 °C) and PLE (ethanol; water, 150 °C, 100 bar). The obtained results were different in terms of the species and methods.

## 7. Conclusions

Literature review included the extractions of phenolic compounds pure carbon dioxide as well as co-solvent modified carbon dioxide. The studies showed a very high interest in polyphenols extraction using supercritical fluid extraction. Pure supercritical carbon dioxide is a safe solvent for this purpose. However, together with ethanol, the extraction efficiency may be enhanced significantly. One should emphasize that the highest extraction yields did not correspond to the highest total phenolic content in all cases. Obviously, co-solvent modified supercritical CO_2_ is more efficient and mostly used in phenolic compounds extraction. The evidence in the number of CO_2_/co-solvent extraction publications representing almost 70% of all described in the review studies, may be a good determinant as well as the confirmation of co-solvent use effectiveness to some extent. The review systematically reflects the issues connected with phenolic compounds extraction. The scattered results from various publications have been gathered into one paper.

According to gathered information in terms of total phenolic content, it is hardly possible to determine which method provided by the authors is the most appropriate for the extraction of particular materials. The task is more complicated if it comes to compare the content of polyphenols in different materials as the authors provided TPC in different units and different equivalents, including mg/g, µg/g, g/kg, mg/ml, µmol/g, weight percentage (wt %) and gallic acid equivalents, catechins equivalent, anthocyanins equivalent and tannic acid, respectively. The most studied was the extraction of grape (seeds, marc, skin, pomace, wastes) and rosemary (leaves).

## 8. Future Recommendations

As it was indicated by a number of publications and reviews, the SFE method did not allow extracting the total content of biologically active phenolic compounds. On the other hand, SFE is not only limited for hydrophilic compounds extraction as a non-polar nature of carbon dioxide may be modified by the addition of polar solvents in order to extract effectively polar groups of compounds. Another aspect is that coupling SFE with other extraction and analytical techniques has gained a lot of interest in recent years. The idea of such techniques makes them a promising tool in the extraction of thermolabile compounds and new directions of researches should be performed towards the optimization of parameters in order to obtain the highest final yield and efficiency. Taking into consideration phenolic compounds extraction with a combined SFE and their importance in various fields, the number of scientific publications is still very sparse.

## Figures and Tables

**Table 1:** Applications of pure CO_2_ in phenolic compounds extraction.

Plant	Part of the Plant	Extraction Conditions			TPC (Total Phenolic Content) *	Reference
		Solvent	Temperature [°C]	Pressure [bar]		
Acai (*Euterpe oleracea*)	berries	CO_2_	50–70	150–490	137.5 mg/100 g (anthocyanins)	[98]
*Achillea millefolium*	leaves	CO_2_	40	100–200	125–152.1 mg/g	[50]
*Baccharis dracunculifolia*	leaves	CO_2_	40–60	200–400	n.d.	[69]
Blackberry (*Rubus* sp.)	bagasse	CO_2_	40–60	150–250	3.31–4.44 mg/g	[70]
Black poplar (*Populus nigra*)	buds	CO_2_	60	300	31.09 µg/mg	[51]
Cranberries	fruits	CO_2_	40	655	n.d.	[66]
Dandelion (*Taraxacum officinale*)	herb	CO_2_	40–60	200–400	n.d.	[45]
*Dorema aucheri*	herb	CO_2_	40–60	150–450	n.d.	[72]
Grape	seeds	CO_2_	35–60	50–150	15.6–22.56 g/kg	[41]
	seeds	CO_2_	75–104	230–538	190–350 mg/g	[40]
	marc	CO_2_	40, 45	100, 120	300.9 mg/g	[99]
	wine lees	CO_2_	40	350	11.9% (*w*/*w*)	[79]
Green tea	leaves	CO_2_	50, 70	100–300	530–578 mg/g	[100]
*Houttuynia cordata*	grass	CO_2_	40	200	n.d.	[78]
Hyssop (*Hyssop officinalis*)	leaves	CO_2_	100	350	n.d.	[33]
Mango (*Magnifera indica*)	leaves	CO_2_	40–50	100–400	n.d.	[82]
*Moringa oleifera*	leaves	CO_2_	40–60	100–200	n.d.	[25]
Olive oil	mill waste	CO_2_	40	350	0.76% (*w*/*w*)	[58]
Peach (*Prunus persica*)	fruits	CO_2_	40–60	100–300	n.d.	[73]
*Phormidium valderianum*	algae	CO_2_	40–60	200–500	117.15 μg/g	[74]
Pomegranate (*Punica granatum*)	leaves	CO_2_	40, 50	100–300	257–389 mg/g	[81]
Purslane (*Portulaca oleracea*)	seeds	CO_2_	60	235	173 mg/g	[71]
Raspberry (*Rubus* sp.)	pomace	CO_2_	30–60	100–450	n.d.	[88]
Rosemary (*Rosmarinus officinalis*)	leaves	CO_2_	40,100	300	n.d.	[80]
		CO_2_	100	350	n.d.	[33]
		CO_2_	50	150–400	33% (*w*/*w*)	[37]
		CO_2_	25–50	80–120	230 mg/g	[85]
		CO_2_	30–40	100–300	n.d.	[76]
		CO_2_	90–110	500–1000	n.d.	[84]
Rye (*Secale cereale*)	bran	CO_2_	30–70	250–550	14.62 mg/g	[89]
Sage (*Salvia officinalis*)	leaves	CO_2_	100	350	n.d.	[33]
		CO_2_	40, 100	300	n.d.	[80]
	herbal dust	CO_2_	40–60	100–300	n.d.	[96]
Spearmint (*Mentha spicata*)	leaves	CO_2_	40–60	100–300	n.d.	[19]
*Strobilanthes crispus*	leaves	CO_2_	40–60	100–200	n.d.	[77]
*Theobroma cacao*	hulls	CO_2_	50	100–200	n.d.	[83]
Thyme (*Thymus vulgaris*)	leaves	CO_2_	100	350	n.d.	[33]
Wheat germ	–	CO_2_	40–60	148–602	6 mg/g extract	[62]
Wine	lees	CO_2_	40	300	11.9% (*w*/*w*)	[79]
Whole flour, medium oat bran, fine bran, low bran	commercial	CO_2_	50	350	n.d.	[87]
Vaccinium meridionale	berries	CO_2_	40, 70	200, 300	n.d.	[97]

* Total phenolic content is provided as gallic acid equivalent, other equivalents than that are provided in the brackets. n.d—no data.

**Table 2:** Applications of co–solvent modified CO_2_ in phenolic compounds extraction.

Plant	Part of the Plant	Extraction Conditions			TPC (Total Phenolic Content) *	Reference
		Solvent	Temperature [°C]	Pressure [bar]		
*Acanthopanax Senticocus*	leaves	CO_2_ + EtOH/H_2_O (80:20, *v*/*v*)	30–60	200–350	n.d.	[27]
*Anacardium occidentale*	leaves	CO_2_, CO_2_ + EtOH (5%, *w*/*w*), CO_2_ + iPrOH (5%, *w*/*w*)	35–55	100–300	n.d.	[91]
Apple	pomace	CO_2_ + EtOH (14–20%, wt %)	40–60	200–600	0.47 mg/g	[4]
*Arrabidaea chica*	leaves	CO_2_ (1 step), CO_2_ + EtOH + H_2_O (2 step)	40	300	178 mg/g	[102]
		CO_2_ (1 step), CO_2_ + EtOH (acidified) (2 step), CO_2_ + H_2_O (acidified) (3 step)	40, 50	300, 400	33–127 mg/g	[103]
Bamboo (*Sasa palmata*)	leaves	CO_2_ + EtOH/H_2_O (5% (25:75, *v*/*v*))	50–110	100–250	7.31 mg/g (catechins)	[104]
Bilberry (*Vaccinium myrtillus*)	press cake	CO_2_ + EtOH/H_2_O (50:50, *v*/*v*, acidified)	50	350	16.67–43.66 mg/g	[105]
	fruits	CO_2_ + EtOH/H_2_O (6, 9%, *w*/*w*)	45	250	n.d.	[52]
Blackberry (*Syzygium cumini*)	fruits	CO_2_ + EtOH	40–60	100–200	n.d.	[106]
Blueberry (*Vaccinium myrtillus)*	pomace	CO_2_ + EtOH	60	80–300	n.d.	[30]
	wastes	CO_2_ + EtOH (5%) + H_2_O (5%)	40	150–250	134 mg/g	[20]
*Bupleurum kaoi*	roots	CO_2_ + EtOH	40	50–200	180–190 μg/g	[107]
*Calycopteris florbunda*	leaves	CO_2_ + EtOH (5%, mass%)	30–50	100–300	n.d.	[47]
Carob (*Ceratonia siliqua*)	pods	CO_2_ + EtOH/H_2_O (10% (80:20, *v*/*v*))	40	220	27.1 mg/g	[108]
Chokeberry (Aronia melanocarpa)	pomace	CO_2_ + EtOH (20, 50, 80, *m*/*m*)	35, 50, 65	75, 100, 125	1.87–1.93 mg/g	[109]
*Citrus paradisi*	peels	CO_2_ + EtOH (5, 10, 15%, wt %)	58.6	95	n.d.	[110]
Coffee	spent ground coffee	CO_2_ + EtOH (5–25%, *w*/*w*)	40–60	350–500	2.99 mg/g	[2]
Cranberries	pomace	CO_2_ + EtOH	60	80–300	n.d.	[30]
Elderberry (*Sambucus nigra*)	berries	CO_2_ + EtOH/H_2_O (50:50, *v*/*v*)	20–60	150–300	60.6 mg/g	[111]
		CO_2_ + EtOH/H_2_O (10% (80:20, *v*/*v*))	40	210	15.8%	[31]
Eucalyptus (*Eucalyptus globulus*)	bark	CO_2_, CO_2_ + EtOH, CO_2_ + EtAc, CO_2_ + H_2_O	50–70	300	57.22 mg/g	[112]
	leaves	CO_2_ + EtOH	35–50	100–200	n.d.	[113]
Gingko (*Ginkgo biloba*)	leaves	CO_2_ + EtOH (5, 10, 12, 24% (mol%))	50–120	242–312	1342 µg/g	[114]
Grape	cane	CO_2_ + EtOH (20%, *v*/*v*)	50	300	n.d.	[106]
	seeds	CO_2_ + MeOH (10, 40%, *v*/*v*), CO_2_ + EtOH (10, 40%, *v*/*v*)	35, 55	350	n.d.	[115]
	seeds	CO_2_ + MeOH (2, 5,10, 15%, *v*/*v*), CO_2_ + EtOH (2, 5, 10, 15%, *v*/*v*)	40	200–300	n.d.	[116]
	seeds	CO_2_, CO_2_ + MeOH (40%)	80	646	n.d.	[117]
	seeds	CO_2_ + EtOH (7%)	37–46	137–167	2.41 mg/g	[118]
	seeds	CO_2_ + EtOH (10, 15, 20%, *w*/*w*)	40	80, 100, 120	7221 mg/g	[7]
	skins, seeds	CO_2_ + EtOH (5–25%, *w*/*w*)	40–60	350–500	2.99 mg/g	[2]
	marc	CO_2_ + EtOH/H_2_O (15% (57:43, *v*/*v*))	40	80–300	2600 mg/g	[119]
	marc	CO_2_ + H_2_O (15%, *w*/*w*), CO_2_ + EtOH (15%, *w*/*w*)	40–60	100, 200	733.6 mg/g	[120]
	marc	CO_2_ + EtOH/H_2_O (10%)	40	80	2736 mg/g	[108]
	skin	CO_2_ + EtOH (7.5%)	40	150	n.d.	[121]
	skin	CO_2_ + EtOH (25–30%)	30–40	100–300	n.d.	[122]
	pomace	CO_2_, CO_2_ + EtOH (8%)	35, 50	80, 350	n.d.	[123]
	pomace	CO_2_ + EtOH (5%, *v*/*v*)	35, 55	100, 400	n.d.	[124]
	by–products	CO_2_, CO_2_ + MeOH (5%, *v*/*v*)	45	150–250	18.1% (*w*/*w*)	[95]
	peels	CO_2_ + EtOH (6–7%)	37–46	157–162	2.156 mg/100 ml	[39]
	bagasse	CO_2_ + EtOH (10%, wt %)	40	200–350	23.0 g/kg	[13]
	by–products, skin, marc	CO_2_ + EtOH	45	200–500	28.12 mg/100g skin	[125]
	wastes	CO_2_, CO_2_ + MeOH (40%)	35	103	n.d.	[17]
	lees from the manufacturing of pisco	CO_2_ + EtOH (10%, wt %)	40	200, 350	2796 mg/kg	[68]
Green algae	–	CO_2_ + EtOH (0–15%, wt %)	40–60	100–300	30.20 mg/g	[126]
Green coffee	beans	CO_2_, CO_2_ + EtOH (5%, *w*/*w*), CO_2_ + iPrOH (5%, *w*/*w*)	50, 60	150, 248, 352	n.d.	[92]
Green propolis	–	CO_2_ + EtOH (1, 2%)	40, 50	250–400	80.3 mg/g	[127]
Green tea	commercial	CO_2_, CO_2_ + H_2_O, CO_2_ + EtOH (18%, 70%, 95%, 99.8%, aq)	50	310	n.d.	[93]
	powdered	CO_2_, CO_2_ +H_2_O	40–60	200–300	n.d.	[128]
	leaves	CO_2_, CO_2_ + EtOH, CO_2_ + EtAc, CO_2_ + EtLac	70	300	6.7 mg/g tea (catechin equivalent)	[129]
Guava (*Psidium guajava*)	seeds	CO_2_ + EtOH (10%, *w*/*w*), CO_2_ + EtAc	50, 60	100–300	n.d.	[46]
Hazelnuts	bug– damaged nuts, rotten nuts	CO_2_ + EtOH (5–25%, *w*/*w*)	40–60	350–500	2.99 mg/g	[2]
Hops (*Humulus lupulus*)	pellets	CO_2_ + EtOH/H_2_O (80:20, *v*/*v*)	50	250	n.d.	[130]
Jatoba (*Hymenaea courbail*)	bark	CO_2_ + H_2_O (9:1, *v*/*v*)	50–60	150–350	335 mg/g (tannic acid equivalent)	[23]
Jucara (*Euterpe edulis)*	residues	CO_2_ + EtOH/H_2_O (50:50, *v*/*v*)	60	200	30 mg/g	[131]
*Koreanum nakai*	leaves	CO_2_ + EtOH (70:30, *v*/*v*)	40–70	200–350	n.d.	[132]
*Maydis stigma*	flowers	CO_2_ + EtOH (20% aq)	40–60	250–450	3.99 mg/g	[5]
*Melia azedarach*	fruits	CO_2_, CO_2_ + EtOH, EtOH, EtOH + H_2_O	50	300	35 mg/g (catechin equivalent)	[133]
*Momordica charantia*	fruits	CO_2_ + EtOH (85% aq)	40–60	250–350	15 mg/g (flavonoids)	[134]
Myrtle (*Myrthus communis*)	leaves	CO_2_, CO_2_ + EtOH (0–30%, wt %)	35–60	100–300	4 μmol/g; 16 μmol/g	[94]
	leaves, berries	CO_2_ + EtOH	45	230	n.d.	[24]
*Odontonema strictum*	leaves	CO_2_ + EtOH (15%)	55, 65	200, 250	99.33–247.78 mg/g	[135]
Oats (*Avena sativa*)	–	CO_2_ + EtOH (80:20, *v*/*v*)	40–70	140–620	1.25 mg/g	[136]
Olive	leaves	CO_2_ + MeOH (10%)	100	334	n.d.	[137]
Orange (*Citrus sinensis*)	pomace	CO_2_, CO_2_ + EtOH (2, 5, 8%, *w*/*w*)	40, 50	100–300	36 mg/g	[59]
*Patrinia villosa*	–	CO_2_ + MeOH (10, 20%)	45–60	150–350	n.d.	[138]
Peach (*Prunus persica*)	pomace	CO_2_ + EtOH (14–20%, wt %)	40–60	200–600	0.26 mg/g	[4]
	leaves	CO_2_ + EtOH (6–20%, wt %)	40–80	150–300	79.92 mg/g	[139]
Pigeonpea (*Cajanus cajan*)	seedlings	CO_2_ + EtOH (80:20, *v*/*v*)	45–65	250–350	n.d.	[44]
Pistachio (*Pistachia vera*)	hulls	CO_2_, CO_2_ + MeOH (5, 15%)	35–55	100–350	7.8 mg/g (tannic acid equivalent)	[140]
Pitanga (*Eugenia uniflora*)	leaves	CO_2_ (1 step), CO_2_ + EtOH (2 step), CO_2_ + H_2_O (3 step)	60	400	240.5 mg/g	[18]
Pomegranate (*Punica granatum*)	seeds	CO_2_ + H_2_O (0, 9, 18 ml/100 g sample), CO_2_ + EtOH (0, 9, 18 ml/100 g sample), CO_2_ + hexane (0, 9, 18 mL/100 g sample)	40–60	200, 275, 350	7.8–72.1 mg/g (tannic acid equivalent)	[26]
Pomelo (*Citrus grandis*)	peels	CO_2_ + EtOH (85:15, *v*/*v*)	60–80	280–420	n.d.	[60]
Puearia lobata	roots	CO_2_ + EtOH (100, 200 mL)	40–60	150–250	173.3 mg/g (flavonoids)	[48]
Purple corn (*Zea mays*)	cob, pericarp	CO_2_ (1 step), CO_2_ + EtOH (2 step), CO_2_ + H_2_O (3 step)	50	400	n.d..	[141]
	cobs	CO_2_ + EtOH/H_2_O (50, 70% aq)	50	400	290 mg/g	[8]
	cob	CO_2_ (1 step), CO_2_ + EtOH (2 step), CO_2_ + H_2_O (3 step)	36–64	259–541	n.d.	[142]
Raspberry (*Rubus* sp.)	pomace	CO_2_ + EtOH	60	80–300	n.d.	[30]
Rosemary (*Rosmarinus officinalis*)	leaves	CO_2_, CO_2_ + EtOH (2%, *v*/*v*)	40–60	300–350	n.d.	[75]
		CO_2_, CO_2_ + EtOH (4, 7%, *v*/*v*)	40–60	150–350	n.d.	[90]
		CO_2_ (1 step), CO_2_ + EtOH (2 step)	40	150, 300	177.61–203.92 mg/g	[143]
		CO_2_ + MeOH (5%)	100	350	160 mg/g	[34]
*Scutellariae Radix*	roots	CO_2_ + MeOH (5, 10, 15%, *v*/*v*)	40–70	200–400	n.d.	[144]
*Solanum stenotomun*	peels	CO_2_, CO_2_ + EtOH (5%, *v*/*v*)	35, 65	100, 400	n.d.	[54]
Soybean	meal	CO_2_ + MeOH /H_2_O (80:20, *v*/*v*)	40–70	300–600	n.d.	[65]
	cake	CO_2_ + EtOH/H_2_O (70:30, *v*/*v*)	50–80	300–400	n.d.	[64]
	expellers	CO_2_ + EtOH	35, 40	400	10.6–16.0 mg/100 g	[145]
Spruce bark (*Picea abies*)	wastes	CO_2_, CO_2_ + EtOH (70:30, *v*/*v*)	40–60	100–200	314.49 mg/g	[57]
Sweet basil (*Ocimum basicilum*)	leaves, roots	CO_2_ + H_2_O (1, 10, 20%, wt %)	30–50	100–300	n.d.	[146]
Tea *(Camelia sinensis)*	seed cake	CO_2_ + EtOH/H_2_O (60:40, *v*/*v*)	40–80	150–450	n.d.	[147]
*Theobroma cacao*	pod husk	CO_2_ + EtOH (13.7%)	40–60	100–300	12.87 mg/g	[148]
*Undaria pinnatifida*	seaweed	CO_2_ + EtOH (3%, *v*/*v*)	30–60	80–300	700 µg/g	[86]
Wine	by–products	CO_2_, CO_2_ + MeOH (5%, *v*/*v*)	45	150–250	18.1% (*w*/*w*)	[95]

* Total phenolic content is provided as gallic acid equivalent, other equivalents than that are provided in the brackets. n.d—no data, EtOH—ethanol, MeOH—methanol, H_2_O—water, EtAc—ethyl acetate, EtLac—ethyl lactate, iPrOH—isopropyl alcohol.
